# Neutrophil Extracellular Traps Form a Barrier between Necrotic and Viable Areas in Acute Abdominal Inflammation

**DOI:** 10.3389/fimmu.2016.00424

**Published:** 2016-10-10

**Authors:** Rostyslav Bilyy, Volodymyr Fedorov, Volodymyr Vovk, Moritz Leppkes, Tetiana Dumych, Valentyna Chopyak, Georg Schett, Martin Herrmann

**Affiliations:** ^1^Friedrich-Alexander-Universität Erlangen-Nürnberg (FAU), Department of Internal Medicine 3 – Rheumatology and Immunology, Universitätsklinikum Erlangen, Erlangen, Germany; ^2^Danylo Halytsky Lviv National Medical University, Lviv, Ukraine; ^3^Lviv Regional Clinical Hospital, Lviv, Ukraine; ^4^Friedrich-Alexander-Universität Erlangen-Nürnberg (FAU), Department of Internal Medicine 1 – Gastoenterology, Universitätsklinikum Erlangen, Erlangen, Germany

**Keywords:** neutrophils, sepsis, inflammation, neutrophil extracellular traps, neutrophil elastase

## Abstract

Neutrophils form neutrophil extracellular traps (NETs) of decondensed DNA and histones that trap and immobilize particulate matter and microbial pathogens like bacteria. NET aggregates reportedly surround and isolate large objects like monosodium urate crystals, which cannot be sufficiently cleared from tissues. In the setting of acute necrotizing pancreatitis, massive tissue necrosis occurs, which is organized as pancreatic pseudocysts (1). In contrast to regular cysts, these pseudocysts are not surrounded by epithelial layers. We hypothesize that, instead, the necrotic areas observed in necrotizing pancreatitis are isolated from the surrounding healthy tissues by aggregated NETs. These may form an alternative, putatively transient barrier, separating necrotic areas from viable tissue. To test this hypothesis, we investigated histological samples from the necropsy material of internal organs of two patients with necrotizing pancreatitis and peritonitis accompanied by multiple organ failure. Tissues including the inflammatory zone were stained with hematoxylin and eosin and evaluated for signs of inflammation. Infiltrating neutrophils and NETs were detected by immunohistochemistry for DNA, neutrophil elastase (NE), and citrullinated histone H3. Interestingly, in severely affected areas of pancreatic necrosis or peritonitis, chromatin stained positive for NE and citrullinated histone H3, and may, therefore, be considered NET-derived. These NET structures formed a layer, which separated the necrotic core from the areas of viable tissue remains. A condensed layer of aggregated NETs, thus, spatially shields and isolates the site of necrosis, thereby limiting the spread of necrosis-associated proinflammatory mediators. We propose that necrotic debris may initiate and/or facilitate the formation of the NET-based surrogate barrier.

## Introduction

Neutrophils are known to produce neutrophil extracellular traps (NETs) of decondensed DNA and histones that trap and immobilize microbial pathogens, e.g., bacteria/fungi (2) or small inert nanoparticles (3). Recent reports on NETosis demonstrate its role in immobilization and sequestering (isolating) foreign objects, which can otherwise not be cleared from the body [e.g., monosodium urate (MSU) crystals during gout (4) or hydrophobic nanoparticles]. Whereas neutrophil extravasation plays an important role in mediating immune response (5) and was reportedly related to inflammatory insults of the invaded tissues (6), it has recently been reported that neutrophils patrol several body tissues that are not inflamed (7), protecting internal organs, exocrine glands, and their ducts from invading intestinal bacteria (8).

In acute necrotizing pancreatitis, massive tissue necrosis occurs in the abdominal cavity. The massive release of intracellular mediators of pancreatic acinar cells determines the degree of systemic toxicity and consequent multi-organ failure and lethality (9). Therefore, immunological mechanisms need to prevent the further spread of necrosis-derived mediators. Formation of NETs was reported in the pancreata and in blood of mice with acute pancreatitis (10). Pancreatic necrosis present early as acute necrotic collections and are consequently organized and separated from adjacent healthy tissues by granulation tissue (“walled-off necrosis”) (11). Even in less severe cases of acute edematous pancreatitis, inflammatory barrier formation may occur and may lead to the formation of pancreatic pseudocysts. In contrast to regular cysts, pseudocysts are not surrounded by an epithelial layer. Thus, we hypothesize that the acute necrotic areas often observed in massive abdominal inflammation are isolated from surrounding healthy tissues by aggregated NETs. These may form a provisionary tissue barrier separating necrotic areas from remains of viable tissue. To check this possible involvement, we analyzed a series of necropsy samples of patients of Lviv Regional Clinical Hospital, who have died from multi-organ failure and systemic toxicity of massive inflammation in the abdominal region.

## Case Presentations

**Figure 1:**
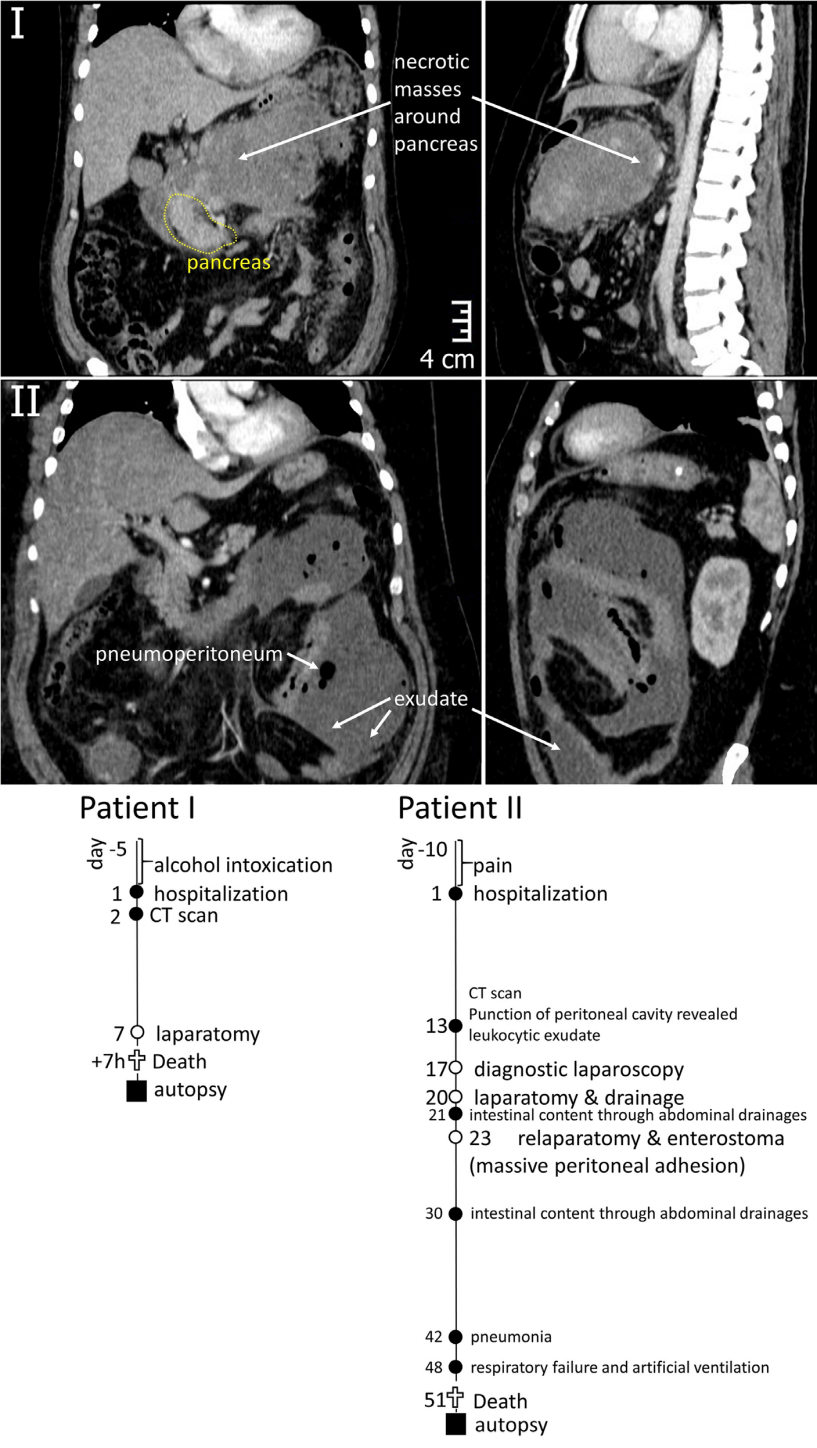
**Top – CT images of the abdominal region of Patients I and II demonstrating the areas of inflammation around pancreas (I) and in peritoneum (II)**. Bottom – graphical representation of disease courses of Patients I & II. Important events are indicated with filled circles, operations – open circles, time of sample collection – filled squares.

Patient I. A 40-year-old man with a history of 5 days alcohol abuse and severe epigastric abdominal pain radiating into the back, with suspect of pancreatitis was hospitalized in December 2014. Upon hospitalization, blood pressure of 110/80 mmHg, heart rate of 152 beats/min (90, hereinafter normal values are represented in square brackets), body temperature of 38.7°C, and respiration rate of 24/min (<20) were recorded. The clinical picture was compatible with systemic inflammatory response syndrome (SIRS). Physical examination revealed that the abdomen was mildly distended with tenderness over the epigastric area. Routine laboratory investigations revealed increased white blood cell count of 14.0 × 10^9^/L (<9.0 × 10^9^/L), as well as levels of pancreatic lipase & amylase, total bilirubin, aspartate transaminase, alanine transaminase, and creatinine, indicating a biliary origin of the pancreatitis and impaired renal function. Levels of procalcitonin and C-reactive protein were increased [1.56 ng/mL (<0.5 ng/mL) and 306.8 mg/L (10 g/L), correspondingly]. A CT scan of the abdomen was performed at day 2 to evaluate the morphological extent of acute pancreatitis (Figure 1). It revealed destructive severe hemorrhagic pancreatitis with signs of necrosis at the body of the pancreas, a necrotic-hemorrhagic mass in the abdomen, ascites, and reactive retroperitoneal lymphadenopathy. Head (49 mm) and tail (39 mm) of the pancreas were clearly differentiated, with no visible pathological inclusions. The patient was admitted to the intensive care unit and managed with aggressive fluid resuscitation and broad-spectrum antibiotics. The patient displayed daily fever. Seven days after hospitalization, the patient underwent laparotomy, necrosectomy, and peripancreatic drainage. Seven hours after surgery, the patient died despite all measures of intensive care due to multiple organ failure.

Patient II. A 50-year-old man was hospitalized in March 2015 with acute pancreatitis. The patient had suffered from pain and dyspepsia for 10 days before hospitalization. Upon hospitalization, blood pressure of 90/60 mmHg, heart rate of 130 beats/min (<90 beats/min), body temperature of 39.1°C, and respiration rate of 22/min (<20) were recorded. Patient displayed nausea and appetite loss, impaired renal function with creatinine of 192 mM/L (<106 mM/L), WBC count of 14.3 × 10^9^/L (<9.0 × 10^9^/L), and highly elevated procalcitonin level of 16.24 ng/mL (<0.5 ng/mL). Ultrasonic examination and blood test for lipase and amylase activity confirmed the diagnosis of acute pancreatitis. The patient also suffered from obesity, diabetes mellitus (glucose level of 17 mM/L on the hospitalization decreasing to 7.7 mM/L upon treatments) and fatty liver disease, with aspartate transaminase level of 68.8 U/L (<31 U/L) and alanine transaminase level of 46.5 U/L (<41 U/L). Thirteen days after hospitalization, the peritoneal cavity contained a leukocytic exudate. A CT scan was made at that time (Figure 1) indicating pneumoperitoneum and inflammatory masses in the pancreatic region. Laparotomy was performed to achieve necrosectomy and drainage, in 1 day (day 21 after hospitalization), intestinal content was recovered from abdominal drainages, which called for repeated surgical interventions (detailed at Figure 1) and massive peritoneal adhesion. Pneumonia have developed at 6th week after hospitalization, causing respiratory failure (day 48). Despite intensive care therapy, multiple organ failure progressed and resulted in the patient’s death at day 51.

## Results

**Figure 2:**
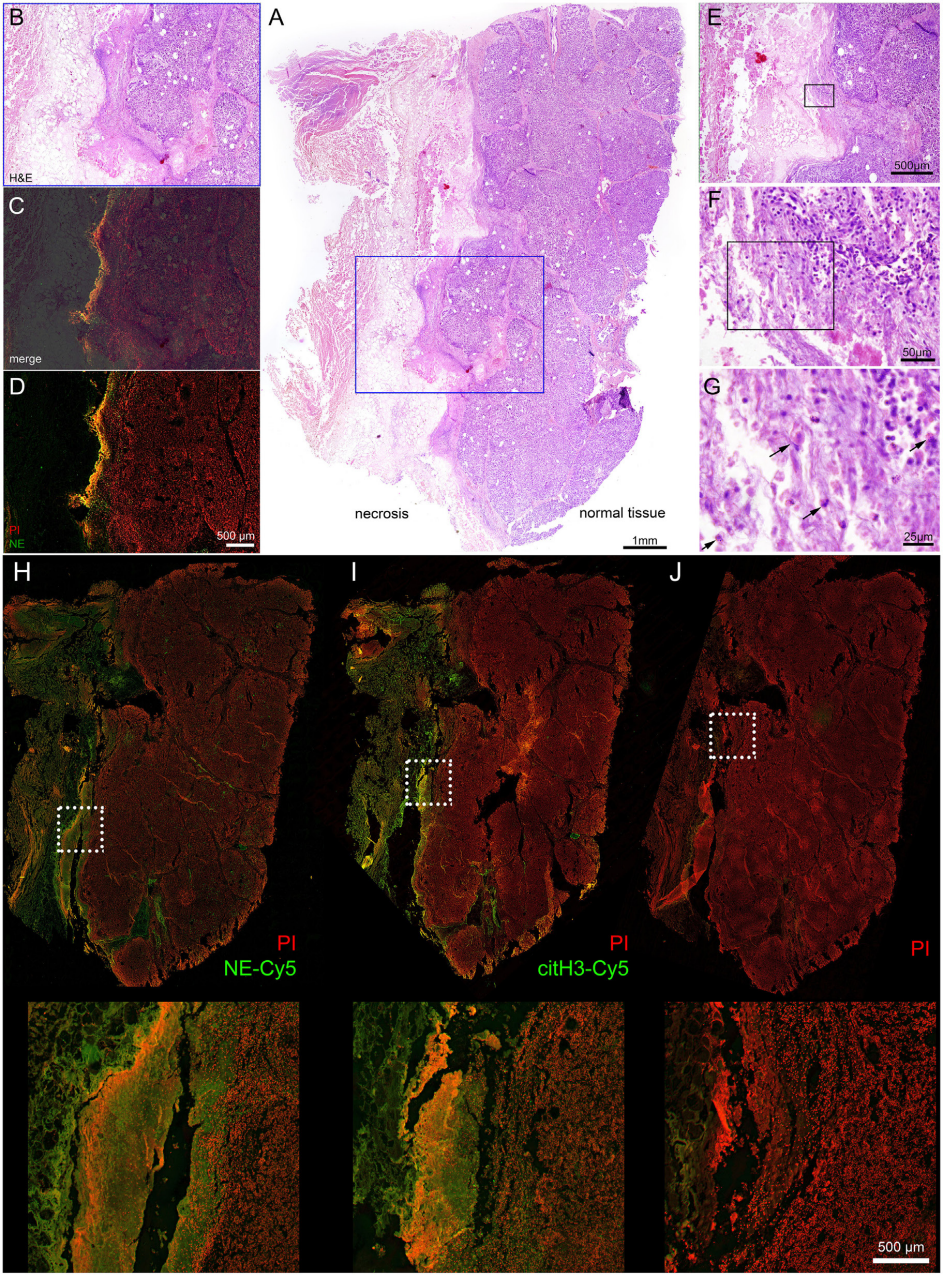
**Interface between normal tissue of the pancreatic gland and necrotic tissue in the area of inflammation, acute pancreatitis**. Patient I. **(A)** – overview (H&E) staining, **(B–D)** – details of region highlighted blue, **(B,E,C)** – merged NE-Cy5 (pseudo colored green) & PI with subsequent slide image stained with H&E, **(D)** – NE-Cy5 and PI, **(E–G)** – sequential details of the indicated areas. **(H)** – immunohistochemistry of histological slide close to **(A)**, stained with NE-Cy5, **(I)** – immunohistochemistry with citH3-Cy5 and PI, **(J)** – secondary Ab and PI staining. The arrows in **(G)** point to cells with swollen nuclei.

**Figure 3:**
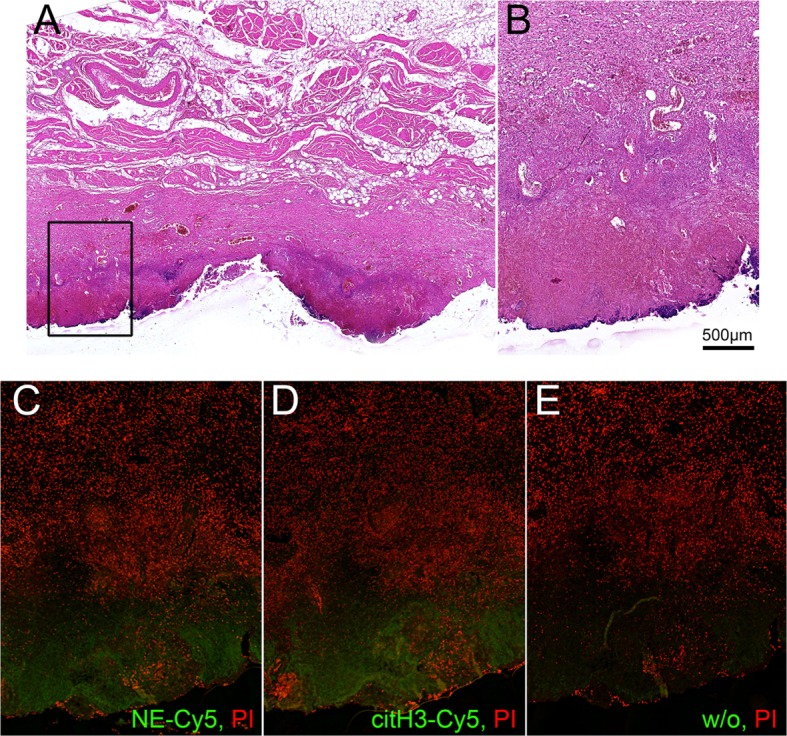
**Interface between normal tissue of peritoneal region gland and necrotic tissue in the area of inflammation**. Patient II. **(A)** – H&E staining, **(B–E)** – detailed area of A, **(C–E)** – immunohistochemistry of adjacent slides, all counterstained with PI, **(C)** – staining for NE-Cy5, **(D)** – staining for citH3-Cy5, **(E)** – secondary Ab.

Necropsy samples of internal organs (19 samples of Patient I and 24 samples of Patient II) were analyzed by hematoxylin and eosin (H&E) staining to reveal areas of the interface between intact and necrotizing tissue. Seven of them were selected and additionally stained for neutrophil elastase (NE) expression. From these samples, we have selected for further detailed analysis the sample of tissue of Patient 1 being the part on the pancreas in the interface between normal tissue and necrosis area (Figure 2) and of Patient II being the part of peritoneum at the interface of healthy and necrotic areas (Figure 3). Both samples were subjected to immunohistochemistry with antibodies against NE and citrullinated histone H3 (citH3) to reveal molecular markers attributable to neutrophils and NETs.

Hematoxylin and eosin staining of the pancreas of Patient I demonstrated that a part of the organ still displayed a regular morphology, while other areas were completely destroyed by necrosis. At the interface between viable and necrotic tissue, the typical histological structure of granulation tissue was to be observed (Figures 2A,B). The superposition of immune histochemistry images for neutrophil elastase staining with H&E staining revealed that the surface of granulation tissues [often referred to as surface leukocyte necrotic layer (12)] is highly positive for NE deposits and DNA (detected by PI staining, detailed at Figure 2J); no regular cellular components were to be detected at this region (Figures 2C,D). Between the marginal zone and viable tissue, high resolution microscopy revealed numerous cellular infiltrates, with nucleus morphology being typical for those of neutrophils. Swelling and release on neutrophil content was also observed (Figures 2E–G). Immunohistochemistry revealed that the mentioned marginal zone was positive for both NE and citH3 signals, while high resolution microscopy demonstrated that areas covering the granulation tissue and displaying neutrophil components were also devoid of cellular material but with abundant amounts of extracellular DNA, judged by the PI signal (Figures 2H–J). High magnification images of this areas are shown on Figure S1 in Supplementary Material, demonstrating different stages of NETosis progression. Thus, these barrier-forming structures possessed typical features of NETs.

Histological analysis of the selected tissue from Patient II also demonstrated the formation of a clear separation zone between the viable tissue of the peritoneal region, mainly composed of muscular fibers and connective tissue, and the zone of necrosis (Figures 3A,B). Deposition of NE and citH3 were clearly seen in this marginal zone followed by the adjacent zone infiltrated with leukocytes that preserved their viable morphology (Figures 2C–E).

## Discussion and Concluding Remarks

Evaluating our data, we propose that the formation of neutrophils extracellular traps serves to form a transient barrier that isolates necrotic tissues during acute inflammatory processes. NETs form in response to necrotic debris and provide a biological sink for damage-associated molecular patterns (DAMPs) by binding to the NET. Potentially harmful DAMPs are then degraded in the NET as previously shown for inflammatory cytokines *via* proteolytic digestion (4). Extracellular DNA and chromatin-associated proteins then contribute to coagulation and reinforce fibrin clots formed at the NET surface (13). This barrier may finally be transformed into fibrotic tissue by slowly migrating and proliferating fibroblasts (14). We have observed abundant NETosis surrounding necrotic areas in two clinical cases of pancreatitis and peritonitis, the abundance of NETosis in other clinical cases, and at different diseases still need further investigations.

The long-term consequences of abundant NETosis surrounding inflammation areas should be taken into account, as products of proteolytic digestion were abundant in pancreatic pseudocysts (15), and the ability of dying cells to modulate immune response was demonstrated (16). As except from cytokine-degrading ability, neutrophil-released enzymes can also possess an ability to modify glycan on the surrounding tissues (17). At the same time, the connection between modification of glycans of IgG molecules and disease activity of lupus was shown (18). Inappropriate clearance of DNA-released material is leading to autoimmune responses, thus connecting NETosis and autoimmunity (19). Abundant production of ROS during neutrophils-induced inflammation is important for the destruction of pathogens, but high levels of ROS in case of massive neutrophil involvement can lead to oxidative stress with ensuing cell death and necrosis (20). Recently, the molecular mechanism of NETosis in response to action to crystals was revealed and shown to include RIPK1-RIPK3-MLKL signaling (21), the involvement of Raf-MEK-ERK pathway was also demonstrated (22); however, it is difficult to check the specific mechanism in the described cases due to massive necrosis and associated release of cellular factors.

Different stages of NETosis, such as early stage of NETosis, progression of NETosis, and aggregated NETs were revealed at histological sections of interfaces between viable and necrotic tissues, as depicted on Figure S2 in Supplementary Material. Staining for DNA (with PI) and for NE allowed the discrimination of those stages. To better understand the different types of cells death in relation to NETosis detection, we summarized the current knowledge of different types of cells death stained for DNA, NE and citH3 in Table 1.

**Table 1:** **NE and DNA localization during different stages of cell death**.

Type of cell death	DNA (PI)	NE	PI-NE colocalization	CitH3
Viable neutrophil	Nuclear	Granular kept inactive by SERPINA1 (23)	No	Few if any
Early NETosis	Cytoplasmic	Cytoplasmic	Yes	Enriched in decondensed chromatin
NETs	Externalized	Externalized bound to DNA in an active form	Yes	Decorating NETs; partially released from chromatin into vicinity of the NETs
Apoptotic cell	Fragmented nuclei	Granular	No	Few if any
Apoptotic body	Apoptotic body	Inside the body	No	Few if any
Secondary necrotic Cell remnant	Fragmented nuclei	Partially released (24)	No	Few if any
Primary necrotic	Nuclear	Released as granula with unknown activity	No	Few if any

Thus, the NETosis role in limiting the spread of necrotic tissues was demonstrated; however, the possible long-term consequences of such process are to be determined and can be connected with frequent complications in patients who suffered from acute inflammation of internal organs.

## Materials and Methods

### Human Samples

Human tissue samples in the form of formalin-fixed paraffin-embedded blocks, obtained as a post mortem biopsies of patients with acute systemic inflammation (sepsis), was obtained from Main Pathoanatomical Laboratory and PathoAnatomical Archieve of Danylo Halytsky Lviv National Medical University upon approval by the Ethics Council of the University. Tissue samples were fixed in formalin and then processed according to the routine H&E staining protocol.

### Computed Tomography

The computed tomography was performed with a multidetector spiral computer tomograph “Aquilion” (Toshiba medical systems, Japan). Exposure doze was 6.0 mSv for Patient I and 5.0 mSv for Patient I. Contrast enhancement with iodine-containing compounds was applied in both cases.

### Histology and Immunohistochemistry

Tissue samples in the form of formalin-fixed paraffin-embedded blocks were used. For general morphology studies, 5–7 μm thick sections were stained with H&E. For immune histochemical analysis, paraffin sections were deparaffinized in toluene–isopropanol–water and incubated for 20 min at 90°C in citrate-containing antigen-retrieval solution. Slides were cooled to room temperature, rinsed in water (2×) and PBS and blocked with 20% of normal goat serum and 2% BSA solution in PBS for 1 h at RT, samples were washed with PBS, and incubated with primary antibodies dissolved in 10% normal goat serum and 2% BSA solution in PBS and incubated overnight at 4°C. The following antibodies were used: rabbit polyclonal antibody to neutrophil elastase (ab68672, Abcam), dilute 1:200 and rabbit polyclonal to histone H3 (citrulline R2 + R8 + R17) – ChIP Grade (ab5103, Abcam). Slides were washed 3× with PBS and incubated with Cy5 AffiniPure Goat Anti-Rabbit IgG (H + L), diluted 1:400 in 10% normal goat serum and 2% BSA solution in PBS and incubated for 1.5 h at 4°C. Slides were washed twice with PBS, counterstained with propidium iodine (2 μg/mL), 10 min at RT, and covered with water soluble fluorescence mounting medium (DAKO).

### Fluorescent Microscopy

Fluorescence microscopy was performed using Keyence bz-x700 robotized microscope using objective 20× NA0.75. Z-stitching was performed for the whole depth of the slide with combining images of maximal sharpness (usually 10 images with 1.0 μm depth), XY-stitching was done for whole slide areas, generating composite image picture. Imaging of PI and Cy5 (pseudo colored green on the presented figures) was done using appropriate filter sets: for PI – OP-87764 with ex. 545/25, em. 605/70, dichroic 565 nm; for Cy5 OP-87766 with ex. 620/60, em. 700/75, dichroic 660 nm. The use of abovementioned filter set allowed simultaneous staining with PI and Cy5 labels.

## Author Contributions

RB, VF, VV, ML, TD, and MH planned experiments and performed most of *in vivo* experiments, conducted data analysis, and wrote the manuscript. VF performed patient’s treatment, operations, and sample collection, arranged, and conducted the CT analyses. VC and GS provided scientific input and wrote the manuscript. RB and MH supervised the project, planned and conducted experiments, data analysis, and wrote the manuscript. All authors read and approved the manuscript.

## Conflict of Interest Statement

The authors declare that the research was conducted in the absence of any commercial or financial relationships that could be construed as a potential conflict of interest.

## Supplementary Material

Figure S1 **Interface between normal tissue of the pancreatic gland and necrotic tissue in the area of acute pancreatitis of Patient I demonstrating different stages of NETosis**. Immunohistochemistry with staining for PI and NE-Cy5.

Figure S2 **Details from the interface area between viable and necrotic tissue of Patient 1, stained with NE & PI, demonstrating colocalization of NE and DNA during the progression of NETosis**. 1–2, 3–4, 5–7, and 8 represent viable neutrophils, early stage of NETosis, progression of NETosis, and aggregated NETs, respectively.
