# Supplementary figures and images for: Neutrophil Extracellular Traps Form a Barrier between Necrotic and Viable Areas in Acute Abdominal Inflammation
Source: Front Immunol. 2016 Oct 10;7:424. doi: 10.3389/fimmu.2016.00424 (PMC5056318; doi:10.3389/fimmu.2016.00424)

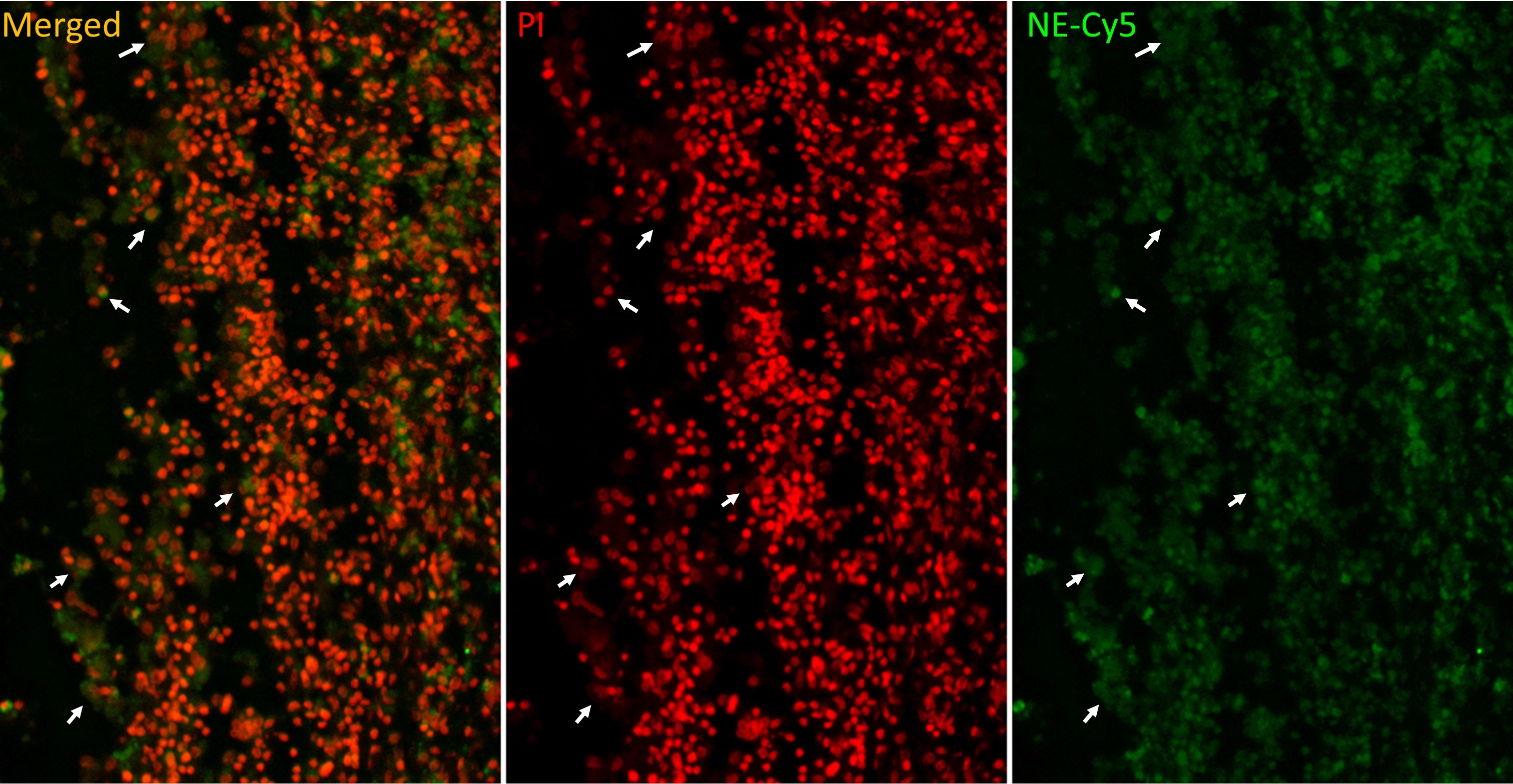

Supplement: Figure S1 — Interface between normal tissue of the pancreatic gland and necrotic tissue in the area of acute pancreatitis of Patient I demonstrating different stages of NETosis. Immunohistochemistry with staining for PI and NE-Cy5. [file image_1.jpeg]

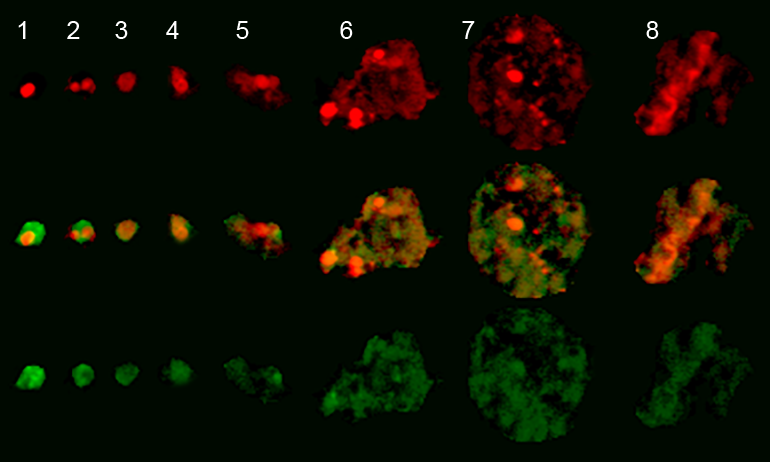

Supplement: Figure S2 — Details from the interface area between viable and necrotic tissue of Patient 1, stained with NE & PI, demonstrating colocalization of NE and DNA during the progression of NETosis. 1–2, 3–4, 5–7, and 8 represent viable neutrophils, early stage of NETosis, progression of NETosis, and aggregated NETs, respectively. [file image_2.tif]
